# The relationship between health literacy and quality of life: a systematic review and meta-analysis

**DOI:** 10.1186/s12955-018-1031-7

**Published:** 2018-10-16

**Authors:** Mengyun Zheng, Hui Jin, Naiyang Shi, Chunxiao Duan, Donglei Wang, Xiaoge Yu, Xiaoning Li

**Affiliations:** 1 Department of Epidemiology and Health Statistics, School of Public Health, Southeast University, Nanjing, 210009 China; 2 Key Laboratory of Environmental Medicine Engineering, Ministry of Education, School of Public Health, Southeast University, Nanjing, 210009 China; 3 Jiangsu Provincial Center for Disease Control and Prevention, Nanjing, 210009 China

**Keywords:** Health literacy, Quality of life, Correlation coefficient, Systematic review, Meta-analysis

## Abstract

**Background:**

Low health literacy often has an association with poor health outcomes such as low levels of self-efficacy, increased mortality, poor health status and reduced quality of life (QOL). The aim of the study was to quantitatively evaluate the relationship between health literacy (HL) and QOL based on a systematic review and meta-analysis.

**Methods:**

EMBASE, PubMed, Web of Science, Elsevier, Cochrane Library, and Chinese electronic databases such as CNKI, and Wanfang were searched from 1970 until February 1, 2018. The pooled correlation coefficient (PCOR) and its 95% confidence interval (CI) between HL and QOL were estimated using R software. Potential sources of heterogeneity were explored using subgroup analysis, sensitivity analysis, and meta-regression.

**Results:**

Twenty-three studies, with a total of 12,303 subjects,were included. The PCOR between HL and QOL was 0.35 (95%CI: 0.25–0.44). Considering different dimensions of HL, the PCOR between QOL and health knowledge, health behavior, health belief, and health skill were 0.36 (95% CI: 0.04–0.61), 0.36 (95%CI: 0.13–0.55), 0.39 (95%CI: 0.10–0.62), and 0.42 (95%CI: 0.03–0.69), respectively. The PCOR between HL and the two dimensions of QOL was lower than the total PCOR between HL and QOL. In subgroup analysis, the PCOR between HL and QOL was 0.46 (95%CI: 0.13, 0.69) among community residents, 0.45 (95%CI: 0.27, 0.61) in China, and 0.45 (95%CI: 0.24, 0.62) based on cohort studies. Sensitivity analyses showed that the stability of results had no significant after excluding the study *(p < 0.001)*. Meta-regression showed that cohort study design, studies conducted in China, and publication before 2012 may be important influencing factors.

**Conclusions:**

Health literacy was moderately correlated with quality of life, but this finding needs to be supported by more evidence.

**Electronic supplementary material:**

The online version of this article (10.1186/s12955-018-1031-7) contains supplementary material, which is available to authorized users.

## Background

Quality of life (QOL) refers to how individuals subjectively assess their own well-being and their ability to perform physical, psychological, and social functions [1]. As an indicator of health and living standards, the concept and connotations of QOL were defined in multiple ways because of different research objectives and purpose, QOL is a multidimensional concept that can fully reflect person’s overall health situation by measuring four dimensions: physical health, physiological health, social health, and mental health. Nowadays, QOL is viewed as a significant outcome of health care and has been increasingly used as a comprehensive health indicator in medical interventions and population health surveys [2]. QOL is mainly used in evaluation of health status and health resources and used as an aspect of influencing factors and health intervention measures, which have higher stability and sensitivity [3].

Health literacy (HL) is linked to literacy and entails people’s knowledge, motivation and competence to access, understand, appraise, and apply health information to make judgments and take decisions in everyday life concerning healthcare, disease prevention and health promotion to maintain or improve QOL during the course of life [4].

At present, the United States, Canada, Australia, and China have all conducted national HL surveys and Europe participated in a comparative European health literacy survey. The survey in Europe showed that there was lack of HL about 47.6% of the study population [5]. In Canada the figure was about 60% [6] and in Australia it was 21% [7], while only 12% in the United States [8], and 9.48% in China had good HL [9]. HL is becoming an important determinant of life expectancy and might also affect QOL.

Many studies [10–15] have investigated the relationship between HL and QOL, but the results seemed inconsistent. Some studies showed that QOL had a positive association with HL, while other studies showed that QOL had a negative association with HL [10, 16]. In China, HL was divided into four dimensions and each was compared QOL: health knowledge, health belief, health behavior and health skill [17–19]. Similarly, the surveys of HL abroad were divided into different levels; QOL differed depending on how high or low the levels of HL were [20–22]. The differences in these results, the survey dimensions of HL and QOL, and the differences in the questionnaires potentially lead to be inconsistent conclusions. Therefore, the aim of this study was to provide a complete overview of the literature regarding the direct impact of HL on QOL, and to discuss the correlation between HL and two dimensions of QOL and the correlation between QOL and four dimensions of HL based on a systematic review and meta- analysis.

## Methods

This systematic review followed the PRISMA (Preferred Reporting Items for Systematic Reviews and Meta-Analyses) guidelines [23] (Additional file 1: Table S1).

We searched English and Chinese language publications on EMBASE, PubMed, Web of Science, Elsevier, Cochrane Library, and Chinese databases such as CNKI and Wanfang from 1970 to February 1, 2018. Studies were searched using logical terms, and search strategy is as follows: “#1 health literacy,” “#2 literacy,” “#3 numeracy”; “#5 quality of life,” “#6 life quality,” “#7 health-related quality of life,” “#8 QOL,” “#9 HRQOL,” “#10 life style.” # 4 is “#1 OR #2 OR #3”, # 11 is “#5 OR #6 OR #7 OR #8 OR #9 OR #10”, and # 12 is “#4 AND #11” finally. Medical subject headings (MeSH) and wild-card options were used where appropriate. Meanwhile, the bibliographies of original studies and reviews were manually searched.

Studies that met the following criteria were included: [1] the study subjects were human, [2] the levels of HL and QOL were conducted and compared as the key objective, [3] the outcome was the correlation coefficient between HL and QOL, and [4] original articles published in English or Chinese (no posters abstracts, letters to the editor etc.).

Studies were excluded for the following reasons: [1] case reports or review articles, [2] QOL or HL was not measured or was not a part of a validated questionnaire, [3] articles were not published in peer-reviewed journals, and [4] there was no the correlation coefficient between HL and QOL. For studies that had been repeated, only the most recent and detailed studies were included in the analysis.

In some studies, there were only QOL scores at different levels of HL without the correlation coefficient between HL and QOL, and there were also some studies with the correlation coefficient compared with the reference materials. These controversial articles are excluded from meta-analysis through discussion.

Two authors independently extracted the following data from the selected studies: the first author, year of publication, design, survey time, location, study population, QOL instrument, and HL instrument. The quality of each of the included studies was independently assessed by two investigators using the Newcastle-Ottawa Scale (NOS) [24] or Agency for Healthcare Research and Quality (AHRQ) [25]. The quality of cross-sectional studies was assessed using AHRQ, and cohort studies were assessed using NOS. The NOS scale has 8 items, and the highest possible total score is 9. The quality of the study is indicated by the scores: 0–5 indicates low quality, 6–7 indicates medium quality, and 8–9 indicates high quality [26]. The AHRQ scale has 11 items, all of which are rated as “yes” (1 point), “no” or “unclear” (0 point), and the highest possible total score is 11. The quality of the study is indicated by the scores: 0–3 indicates low quality, 4–7 indicates medium quality, and 8–11 indicates high quality [27].

### Statistical analysis

Different results indicated effects of different sizes regarding the relationship between HL and QOL. Summary statistics were then calculated. Most meta-analysis did not directly use the values of each correlation coefficients when combined correlation coefficients, because the variance of each correlation coefficient was too dependent on the correlation. Thus, we needed to convert various data into correlation coefficient uniformly, for which meta-analysis was performed. The method we used was to carry out the correlation coefficient (r value) of each study by Fisher’s Z transformation, calculate the standard error, and calculate the summary Fisher’s Z value using the inverse variance. Then a formula was used to transform the Z value into an r value [28].

To calculate the summary or pooled r, and 95% CI, the hypothesis test was used to judge whether the correlation was statistically significant. Lastly, a forest plot was used to indicate the effect size. Publication bias was assessed by funnel plots and the Egger’s test.

R software (R × 64 3.4.1) was used for meta-analysis. Firstly, it estimated heterogeneity between studies using *Q* and *I*^*2*^ statistics. According to the *Q*-statistic, if the *p* < 0.05 and *I*^*2*^ < 50%, it indicated heterogeneity in the risk factors between studies, and the random effect model was used for the meta-analysis. Otherwise, the fixed effect model was used.

In addition, subgroup analysis, sensitivity analysis and meta-regression analysis were conducted through R software. A sensitivity analysis was performed to ensure the stability of the results. Meta-regression analyses were conducted to assess heterogeneity; it can also be used to analyze the differences of categorical explanatory variables introduced in subgroup analysis. The dependent variable of meta-regression is the correlation coefficient between HL and QOL, and the research sample, research type, research population, area, time, research quality and questionnaire type are independent variables. The regression coefficient estimates how the intervention effects of each subgroup differ from the specified reference subgroup. The *p* value < 0.05 of each regression coefficient was considered as statistically significant.

## Results

### Characteristics of eligible studies on the relation between HL and QOL

**Fig. 1:**
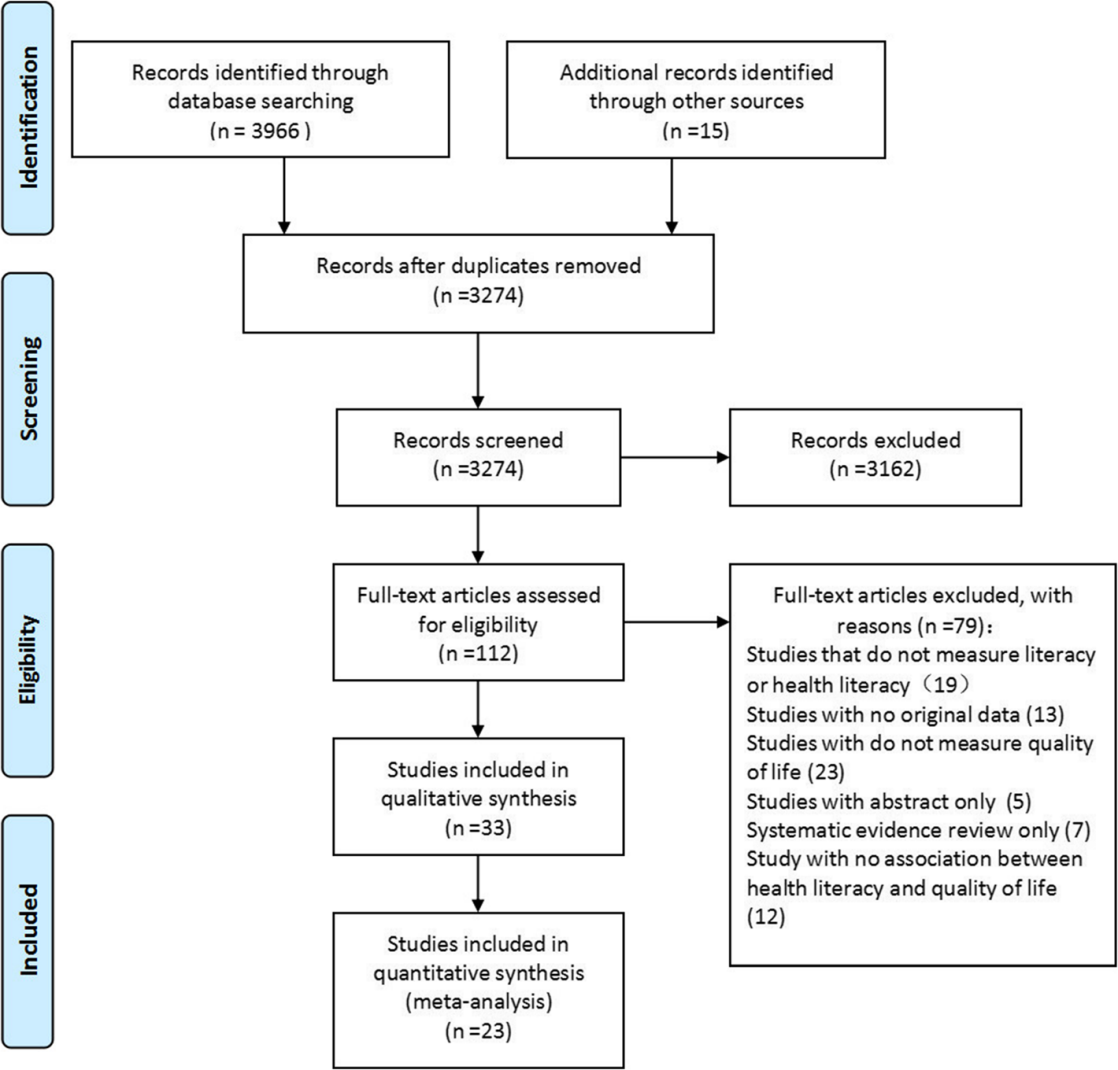
Flowchart of study selection

Based on their titles and abstracts, 3274 articles met our criteria. After careful review, 23 studies [11–13, 16–20, 29–43] fulfilled our selection criteria for meta-analysis. The flow chart of this selection procedure is shown in Fig. 1

.

The included studies were published between 2005 and February 2018. The detailed information for review is described in Table 1. The design types of these studies included cohort studies and cross-sectional studies. A total of 13 studies were conducted between 1995 and 2015, but 10 studies had no survey time attributed to them. The study subjects were mainly college students, community residents and patients. They were mostly adults, and the majority of them were women. The instruments of measuring QOL were mainly the EuroQol-5 Dimension (EQ-5D) [44], the 12 item short form health questionnaire survey (SF-12), the 36 item short form health questionnaire survey (SF-36) [45] and other self-developed questionnaires, while the instruments of HL were mainly the Test of Functional Health literacy in Adults (TOFHLA) [46], the Rapid Estimate of Adult Literacy in Medicine (REALM) [47], the Newcastle-Ottawa Scale (NVS) [48], and self-developed questionnaires. The details are shown in Table 1.

**Table 1:** Summary of the 23 publications included in the review

Author	Design	Survey time	Location	Study population	QOL instrument	HL instrument	Quality score
Chunhua L, et al. 2013 [17]	Cross-sectional	April–June, 2012	Guangzhou,China	2109 college students (age: 15–28 years, 55.2% female)	EQ-5D	Self-developed scales: Three aspects of HL, Five types of HL	9
Nan W, et al. 2012 [18]	Cross-sectional	–	Jiaozuo, China	600 community elders (age: over 45 years, 57.5% female)	SF-36	Questionnaire on the health literacy of Chinese citizens	6
Liu L, et al. 2016 [19]	Cross-sectional	January–September, 2015	Urumqi,China	556 coronary heart disease patients (age: 45–83 years, 31.1% female)	EQ-5D	Self-developed questionnaire containing Four dimensions of HL: knowledge, attitude, behaviors, skills.	9
Yan Z, et al. 2012 [43]	Cross-sectional	–	Jilin, China	192 empty nest elders(over 65 years, 57.8% female)	SF-36	Self-developed questionnaire measuring health literacy of elderly	6
Qiyuan L, et al. 2011 [42]	Cross-sectional	May–November, 2010.	Yanji, China	331 Hypertensive elderly (over 60 years, 63.4% female)	Self-developed questionnaire containing Global QOL, Psychological general well-being, Symptom bother	Self-developed questionnaire measuring health literacy about hypertension	9
Wenning D, et al. 2015 [41]	Cross-sectional	November–December, 2014.	Kunming, China	500 college students (age: 17–24 years, 56.7% female)	Self-developed questionnaire measuring QOL of college students	Self-developed questionnaire measuring health literacy of college students	8
Couture EM, et al. 2017 [34]	Cross-sectional	–	Quebec, Canada	247 chronic Participants (age: 18–85 years, 55.5% female)	SF-12v2	NVS	7
Halverson JL, et al. 2015 [35]	Cross-sectional	2006s-sect	Wisconsin, America	1841 Wisconsin residents, newly diagnosed with lung, prostate, breast, or colorectal cancer(age: over 18 years, 50.8% female)	FACT-G	Self-developed questionnaire containing four questions validated in STOFHLA and REALM	8
Naimi AJ, et al. 2017 [11]	Cross-sectional	–	Tehran, Iran	400 hypertensive patients (age: 18–89 years, 45.0% female)	SF-36	HELIA	6
Song S, et al. 2017 [38]	Cross-sectional	October ectionale patie	South Korea	305 non-institutionalized adults (age: 20–60 years, 50.5% female)	SF-36	REALM	6
Wang C, et al. 2015 [31]	Cross-sectional	–	Northwestern China	913 poor rural women (age: 23–57)	EQ-5D	R-CAHLQ	6
Wang C, et al. 2017 [13]	Cross-sectional	2001s-sec	Six towns,China	882 hypertensive patients (age: over 35 years, 56.1% female)	SF-36	Self-developed questionnaire validated three-item BHLS	7
Rocha PC, et al. 2017 [37]	Cross-sectional	–	Belo Horizonte,Brazil	384 adolescents (age: 15 and 19 years, 70.3% female).	PedsQL	Self-developed questionnaire, composed of closed questions	5
Macabasco OA, et al. 2011 [30]	Cross-sectional	2007–2009	America	605 patients with symptomatic heart failure (age: over 18 years, 48.0% female)	HFSS	TOFHLA	7
Johnston MV, et al. 2005 [36]	Cross-sectional	–	new jersey,America	107 patients with spinal cord injury (age: over 18 years, 17.8% female)	SF-12,SWLS	TOFHLA	6
Ownby RL, et al. 2014 [12]	Cross-sectional	–	Central and South America as well as Mexico and the US	475 English- and Spanish-speaking community-dwelling volunteers(age: 18–81 years, 60% female)	SF-36,EQ-5D	TOFHLA, REALM	5
Zhang XH, et al. 2009 [40]	Cross-sectional	–	a tertiary referral center,Singapore	199 patients with rheumatic diseases (over 18 years, 70.5% female)	SF-36,EQ-5D, and SF-6D.	REALM	6
Wallace LS, et al. 2008 [39]	Cross-sectional	September, 2004	Tennesseans,America	249 patients (age: over 18 years, 65.1% female)	Self-developed: fouritems developed and validated by CDC	REALM	8
Son YJ, et al. 2016 [32]	Longitudinal cohort	June 2012–July 2013	Cheonan,Korean	238 PCI patients (33.2% female)	Self-developed: a validating 10-item questionnaire	Self-developed containing the three-item set of brief screening questions	9
Mancuso CA, et al. 2006 [33]	Longitudinal cohort	1995–1999	New York,America	175 asthma patients (mean age: 40 years, 83% female)	AQLQ	TOFHLA	9
Al SF, et al. 2016 [29]	Longitudinal cohort	December 2011–December 2013	Alberta,Canada	1948 Patients with type 2 diabetes (age: over 18 years, 45.0% female)	EQ-5D,SF-36	BHLS	7
Montbleau KE, et al. 2017 [10]	Cohort	–	an urban, safety-net hospital,America	40 patients with Atrial fibrillation (age: over 60 years, 45.0% female)	SF-36	STOFHLA	6
Husson O, et al. 2015 [20]	Longitudinal	2000–2009	southern part of the Netherlands	1626 Colorectal cancer survivors (age: over 18 years, 42.9% female)	EORTC QLQ-C30	SBSQ	9

*EQ-5D* the European Quality of Life-5 Dimensions, *SF-36* the 36-item Short Form, *SF-12v2* Short Form Health Survey, *NVS* the Newest Vital Sign, *FACT-G* the Functional Assessment of Cancer Therapy-General, *STOFHLA* Short Test of Functional Health Literacy in Adults, *REALM* the Rapid Estimate of Adult Literacy in Medicine, *HELIA* Health Literacy for Iranian Adults, *R-CAHLQ* the revised Chinese Adult Health Literacy Questionnaire, *BHLS* Brief Health Literacy Screening, *PedsQL* the Paediatric Questionnaire on Quality of Life, *HFSS* the Heart Failure Symptom Scale, *TOFHLA* Test of Functional Health Literacy in Adults, *SWLS* Satisfaction with Life Scale**,** *AQLQ* the Asthma Quality of Life Questionnaire, *SBSQ* Chew’s three-item Set of Brief Screening Questions, *EORTC QLQ-C30* Research and Treatment of Cancer Quality of Life Questionnaire C30

### Methodological quality

NOS was used to score 4 of the cohort studies, and 19 of the cross-sectional studies were scored using the AHRQ. Of the studies scored by NOS, the minimum score was 7, the maximum score was 9, and the average score was 8.5. Of the studies scored by AHRQ, the minimum score was 5, the maximum score was 9, and the average was 6.89 (Table 1).

### The correlation between HL and QOL

#### General correlation

**Fig. 2:**
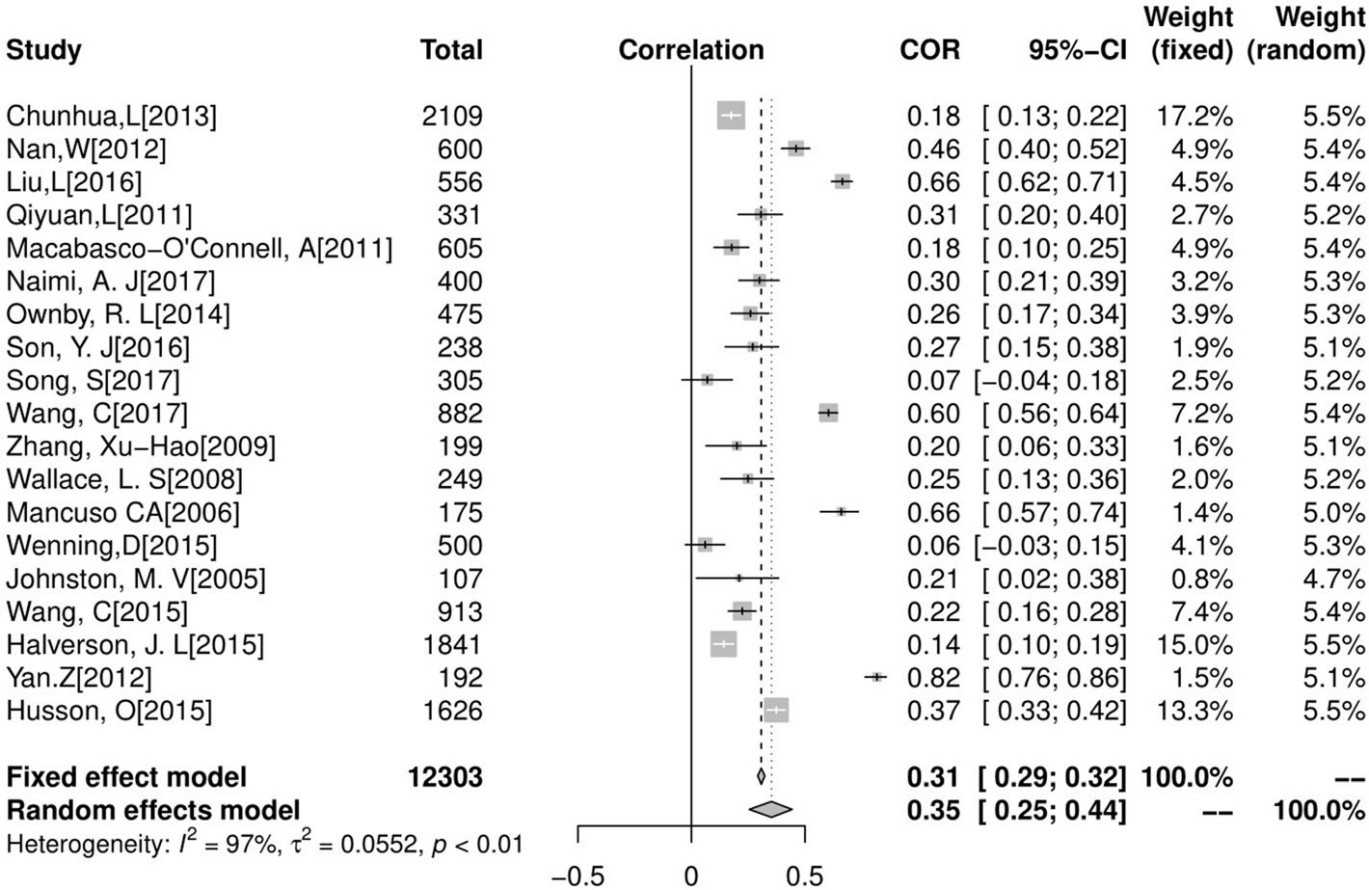
Forest plot of the correlation between HL and QOL

A total of 19 studies were included in the analysis of the correlation between HL and QOL, and the total sample size was 12,303. In the heterogeneity test: the correlation between HL and QOL (*I*^*2*^ *= 97%*, *p < 0.001*) showed that there was heterogeneity. The correlation coefficient between QOL and HL was 0. 35 (95% CI: 0.25–0.44) (Fig. 2)

.

### The correlation between HL and two dimensions of QOL

The QOL included physical and mental scores. A total of 8 studies were included in the analysis of the correlation between HL and physical QOL, and the total sample size was 5777. In the heterogeneity test, the correlation between HL and physical QOL (*I*^*2*^ = 94%, *p*<*0.001*) showed that there was heterogeneity, using a random effect model to combine effect quantity. The correlation coefficient between physical QOL and HL was 0. 20 (95% CI: 0.08–0.31) (Fig. 2).

In addition, a total of 7 studies were included in the analysis of the correlation between HL and mental QOL, and the total sample size was 5602. In the heterogeneity test, the correlation between HL and mental QOL (*I*^*2*^ = *95%*, *p*<*0.001*) showed that there was heterogeneity, using a random effect model to combine effect quantity. The correlation coefficient between mental QOL and HL was 0. 18 (95% CI: 0.08–0.31).

### The correlation between QOL and four dimensions of HL

HL incorporates health knowledge, health behavior, health belief and health skill. A total of 3 studies were included in the analysis of the correlation between QOL and four dimensions of HL, and the total sample size was 3256. In the heterogeneity test, the correlation between health knowledge of HL and QOL (*I*^*2*^ *= 99%, p<0.001*) showed that there was heterogeneity. The correlation coefficient between QOL and health knowledge was 0.36 (95% CI: 0.04–0.61). In the heterogeneity test, the correlation between health behavior of HL and QOL (*I*^*2*^ = 97%, *P*<*0.001*) showed that there was heterogeneity, using a random effect model to combine effect quantity. The correlation coefficient between QOL and health behavior was 0.36 (95% CI: 0.13–0.55). In the heterogeneity test, the correlation between health belief of HL and QOL (*I*^*2*^ *= 98%*, *p*<*0.001*) showed that there was heterogeneity. The correlation coefficient between QOL and health belief was 0.39 (95% CI: 0.10–0.62). In the heterogeneity test, the correlation between health skill of HL and QOL (*I*^*2*^ *= 99%*, *p*<*0.001*) showed that there was heterogeneity. The correlation coefficient between QOL and health skill was 0.42 (95% CI: 0.03–0.69).

### Subgroup analysis

The subgroup analysis included population, time, study design, area, study quality and the kinds of HL instruments and QOL instruments used (Table 2). Noticeably, the correlation coefficient between HL and QOL was 0.46 (95%CI: 0.13, 0.69) among community residents, 0.45 (95%CI: 0.27, 0.61) in China, and 0.45 (95%CI: 0.24, 0.62) based on cohort study design. The correlation coefficient between HL and QOL on TOFHLA was higher than REALM.

**Table 2:** Subgroup analysis of the correlation between HL and QOL

Subgroup	Sample size	No. of studies	correlation coefficient [95%CI]	P
Total	12,303	19	0.35 [0.25; 0.44]	< 0.01
PopulationI				
College students	2609	2	0.12 [0.01; 0.23]	0.02
Community residents	1572	4	0.46 [0.13; 0.69]	< 0.01
PopulationII				
Patients	8122	13	0.35 [0.25; 0.44]	< 0.01
Health	4181	6	0.35 [0.14; 0.53]	< 0.01
Nation				
China	6083	8	0.45[0.27; 0.61]	< 0.01
America	3452	6	0.29 [0.16; 0.42]	< 0.01
Other Asian countries	1142	4	0.21 [0.10; 0.32]	0.01
HL questionnaire				
REALM	753	3	0.17 [0.06; 0.28]	0.08
TOFHLA	1362	4	0.35 [0.12; 0.54]	< 0.01
Other instruments	10,188	12	0.39 [0.27; 0.51]	< 0.01
QOL questionnaire				
EQ-5D	2665	2	0.45 [0; 0.80]	< 0.01
SF	4073	9	0.38 [0.22; 0.53]	< 0.01
Other instruments	5565	8	0.29 [0.17; 0.39]	< 0.01
Study design				
Cross-sectional	10,264	16	0.33 [0.22; 0.44]	< 0.01
Cohort	2039	3	0.45 [0.24; 0.62]	< 0.01
Study quality				
High	7625	9	0.34 [0.20; 0.46]	< 0.01
Medium	4678	10	0.36 [0.21; 0.50]	< 0.01
Low	0	0		
Time				
Before 2012.01	5709	7	0.37 [0.21; 0.51]	< 0.01
After 2012.01	3708	5	0.27 [0.01; 0.49]	< 0.01
No found	2886	7	0.39 [0.21; 0.54]	< 0.01

### Publication bias

**Fig. 3:**
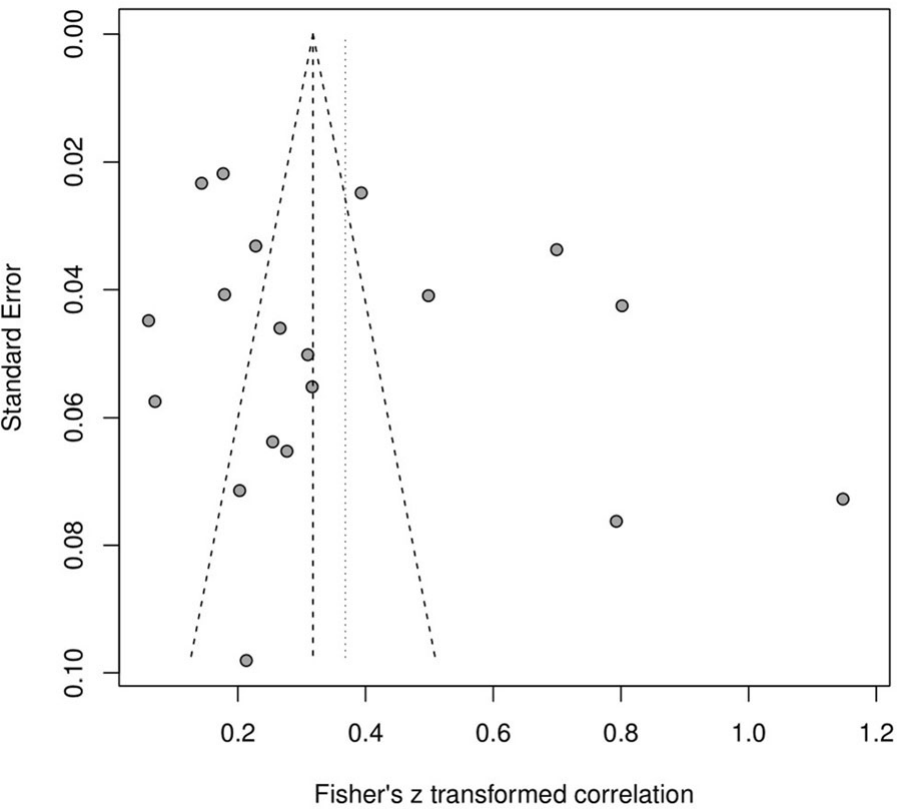
Funnel plot of the correlation between HL and QOL

Funnel plot asymmetry was observed in the studies of correlation between HL and QOL (Fig. 3)

**Fig. 4:**
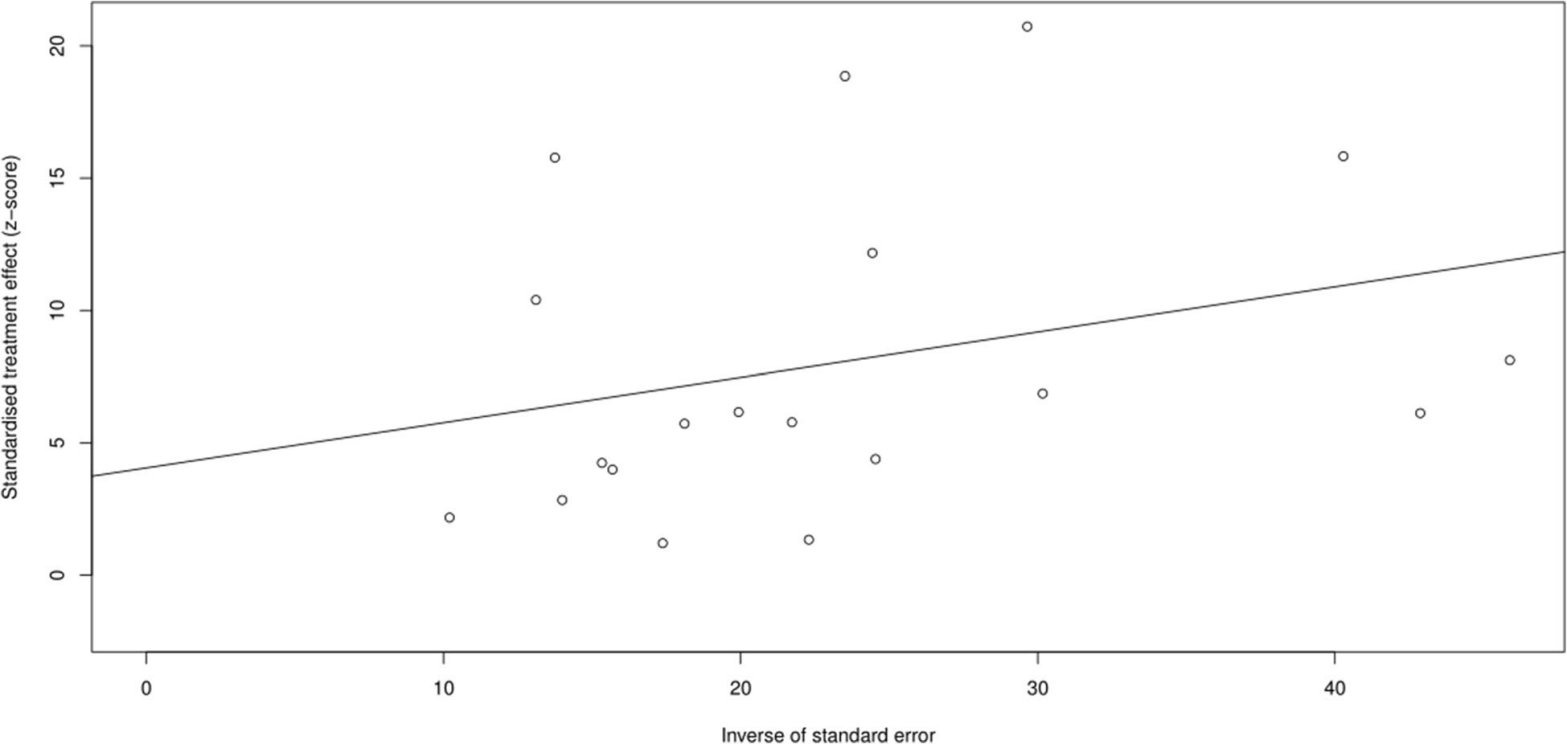
Egger test

. Egger’s test (*t* = 1.197, *p* = 0.248) indicated that there was no obvious publication bias (Fig. 4)

.

### Sensitivity and meta-regression analysis

Sensitivity analyses were used to evaluate the effect of each study on the pooled results by sequentially excluding single studies. It was found that the results had no significant change after excluding each study (Additional file 2: Table S2). Meta-regression analysis was used to detect heterogeneity (Table 3). The results showed that cohort study design, studies conducted in China and publication before 2012 may be important influencing factors.

**Table 3:** Meta regression on correlation coefficient

Variables		Estimate	95%CI	*P*-value
Intercept		0.93	[0.37 1.49]	0.001
Sample size		−0.00	[−0.00–0.00]	0.000
Design	Cross-sectional	ref		
	Cohort	0.47	[0.11 0.83]	0.011
Area	America	Ref		
	China	0.37	[0.06 0.69]	0.021
	Asia other country	0.26	[−0.18 0.72]	0.246
Population	Health	ref		
	Patient	−0.25	[−0.55 0.05]	0.097
HL questionnaires	TOFHLA	ref		
	REALM	−0.41	[−0.85 0.02]	0.059
QOL questionnaires	EQ-5D	ref		
	SF	−0.74	[−1.50 0.01]	0.053
Study quality	High	ref		
	medium	0.22	[−0.34 0.77]	0.443
Time	After 2012	ref		
	Before 2012	0.61	[0.12 1.09]	0.014

## Discussion

With the development of medical technology and the increase of life expectancy, people pay more attention to their QOL. Health managers and researchers are more concerned about whether improvements in health literacy increase people’s QOL. Song, S. [38] showed HL was not positively correlated with QOL, and the correlation coefficient between HL and QOL was only 0.07. However, Liu, L [19] showed that the correlation coefficient between HL and QOL among patients with coronary heart disease was 0.665. This study showed the HL had a moderate positive correlation with QOL (*r* = 0.35, *p* < 0.05) through meta-analysis, which is helpful for further research. It suggested that people with low HL may pay low attention to their health status and therefore they had unhealthy behavior habits that caused a decline of QOL [49]. This study also analyzes the interaction relations between dimensions of HL and dimensions of QOL. The correlation between HL and the two dimensions of QOL was lower than the total correlation coefficient of overall HL and QOL. The correlation between QOL and the four dimensions of HL was higher than the total correlation coefficient of overall HL and QOL, among which the correlation between health skills and QOL was highest. Health skill refers to the ability of individuals to transform health knowledge into healthy behaviors. It plays an intermediary role between health knowledge and health behavior. Good health skills improve health status and QOL. Mental health conditions closely related to the quality of the individual’s life will also improve health skills. Some studies divided the QOL into two dimensions [29, 34, 36], while other studies divided it into more than two dimensions [20, 50]. However, after HL and QOL were divided into different dimensions, fewer studies were included as they may not be general and reliable correlation coefficients.

In subgroup analysis, population, time, study design, study quality, area, and the type of HL instruments and QOL instruments were analyzed. Among the population subgroup, the college students’ correlation coefficient between HL and QOL was lower, it may be that the overall health literacy of college students is similar, but the difference in quality of life scores is more related to the psychological status of students such as anxiety and depression. In addition, the patients’ correlation coefficient was higher than that of the healthy population, indicating that patients who had higher health knowledge had relatively low requirements for QOL. In terms of study design, the cohort study’s correlation coefficient between HL and QOL was higher than cross-sectional study’s correlation due to the study design. In terms of region, the correlation coefficient of studies conducted in China was the highest, followed by American region and other regions of Asia. The reason may be that the sample size of China was 6083: higher than the United States (3452) and other parts of Asia (1142). For the instruments, TOFHLA for the HL questionnaire and the short form questionnaire for the QOL questionnaire were better than others. Similar to the result of subgroup analysis, meta-regression analysis also showed that cohort study design, studies conducted in China, and publication before 2012 may be important influencing factors.

This study has some limitations. The first limitation is in the study design used. Mainly cross-sectional studies were included, which collected HL and QOL at the same time and never reflected on the long-term impact of lower HL on QOL. This is why the cohort study’s correlation coefficient between HL and QOL was higher than cross-sectional study’s correlation, and the insufficiency is that there are fewer studies included in the cohort study. The second limitation is with regard to the quality of the data collected. The data of studies included *β* and r and rank correlation r. Some studies [21, 31, 50, 51] showed that compared poor and medium HL with high HL showed that β was not the general value between HL and QOL. As a result, this part of the value was not included in the meta-analysis. The third limitation is with regard to the questionnaire used. The questionnaires for HL and QOL were not unified, which increased the heterogeneity of the meta-analysis and the subsequent possibility of bias in the results. Therefore, studies with larger sample sizes and better data quality are needed to further confirm the finding. In addition, the heterogeneity of the subjects, the different ages and health conditions are influencing factors on the results.

## Conclusion

In summary, HL was moderately correlated with QOL, and the correlation coefficient between QOL and health knowledge, health behavior, health belief, and health skill were statistically significant. However, these findings need to be supported by more evidence.

## Additional files

Additional file 1: **Table S1.** PRISMA checklist. (DOC 61 kb)

Additional file 2: **Table S2.** Sensitivity analysis of Meta. (DOCX 15 kb)

## Abbreviations

AHRQ: Agency for Healthcare Research and Quality
CI: Confidence Interval
CNKI: China National Knowledge Infrastructure
EQ-5D: The European Quality of Life-5 Dimensions
HL: Health Literacy
MeSH: Medical subject headings
NOS: The Newcastle-Ottawa Scale
NVS: The Newest Vital Sign
PCOR: The pooled correlation coefficient
QOL: Quality of Life
REALM: The Rapid Estimate of Adult Literacy in Medicine
SF-12: The 12-item Short Form
SF-36: The 36-item Short Form
TOFHLA: Test of Functional Health Literacy in Adults
