# Supplementary material for: The relationship between health literacy and quality of life: a systematic review and meta-analysis
Source: Health Qual Life Outcomes. 2018 Oct 16;16:201. doi: 10.1186/s12955-018-1031-7 (PMC6192335; doi:10.1186/s12955-018-1031-7)
Supplement: Supplementary file 1 — Table S1. PRISMA checklist. (DOC 61 kb) [file 12955_2018_1031_MOESM1_ESM.doc]

| **Section/topic** | **#** | **Checklist item** | **Reported on page #** |
| --- | --- | --- | --- |
| **TITLE** | | |  |
| Title | 1 | The report is identified as a systematic review and meta-analysis. | 1 |
| **ABSTRACT** | | |  |
| Structured summary | 2 | The structured abstract includes Background, Methods, Results, Conclusion | 2-3 |
| **INTRODUCTION** | | |  |
| Rationale | 3 | Describe in the introduction | 4-5 |
| Objectives | 4 | Stated the PICO in the introduction | 4-5 |
| **METHODS** | | |  |
| Protocol and registration | 5 | The protocol is described in the Methods. Registration does not apply. | NO |
| Eligibility criteria | 6 | Specify study characteristics and report characteristics used as criteria for eligibility and rationale are described in the Methods | 5-6 |
| Information sources | 7 | All information sources in the search and date last searched are described in the Methods | 5 |
| Search | 8 | The search strategy are present in the Methods | 5-6 |
| Study selection | 9 | The process for selecting studies are stated in the Methods | 5-6 |
| Data collection process | 10 | The method of data extraction from studies are described in the Methods | 6 |
| Data items | 11 | List and define some variables for which data were sought and any assumptions and simplifications made in the Methods. | 6-7 |
| Risk of bias in individual studies | 12 | Describe methods used for assessing risk of bias of individual studies (including specification of whether this was done at the study or outcome level), and how this information is to be used in any data synthesis. | 7 |
| Summary measures | 13 | the principal summary measures are stated in the Methods | 6-7 |
| Synthesis of results | 14 | the methods of handling data and combining results of studies are described in the Methods | 6-8 |

Page 1 of 2

| **Section/topic** | **#** | **Checklist item** | **Reported on page #** |
| --- | --- | --- | --- |
| Risk of bias across studies | 15 | Specify the assessment of risk of publication bias in the Methods | 7-8 |
| Additional analyses | 16 | Describe methods of sensitivity or subgroup analyses and that were pre-specified in the Methods | 7-8 |
| **RESULTS** | | |  |
| Study selection | 17 | The numbers of studies screened, assessed for eligibility and a flow diagram are given in the result. | 8 |
| Study characteristics | 18 | For each study, characteristics for which data were extracted and provide the citations are present in the result. | 8 |
| Risk of bias within studies | 19 | Present data on risk of bias of each study and, if available, any outcome level assessment (see item 12). | 10-11 |
| Results of individual studies | 20 | for each study: (a) simple summary data for each group (b) effect estimates and confidence intervals with a forest plot are present in the result. | 8-10 |
| Synthesis of results | 21 | The results of meta-analysis including confidence intervals and measures of consistency are present in the result. | 9-10 |
| Risk of bias across studies | 22 | The results of assessment of risk of publication bias across studies are present in the result. | 10-11 |
| Additional analysis | 23 | Give results of sensitivity and subgroup analyses in the result. | 10-11 |
| **DISCUSSION** | | |  |
| Summary of evidence | 24 | The main findings including the strength of evidence for each main outcome are summarized in the discussion. | 11-13 |
| Limitations | 25 | Limitations about this meta-analysis are discussed in the discussion. | 13 |
| Conclusions | 26 | The result of the study, and implications for future research are provided in the discussion. | 13 |
| **FUNDING** | | |  |
| Funding | 27 | Sources of funding for the meta-analysis are described in the Acknowledgements. | 14 |

*From:*  Moher D, Liberati A, Tetzlaff J, Altman DG, The PRISMA Group (2009). Preferred Reporting Items for Systematic Reviews and Meta-Analyses: The PRISMA Statement. PLoS Med 6(6): e1000097. doi:10.1371/journal.pmed1000097

For more information, visit: **www.prisma-statement.org**.

Page 2 of 2
