# Supplementary material for: The relationship between health literacy and quality of life: a systematic review and meta-analysis
Source: Health Qual Life Outcomes. 2018 Oct 16;16:201. doi: 10.1186/s12955-018-1031-7 (PMC6192335; doi:10.1186/s12955-018-1031-7)
Supplement: Supplementary file 2 — Table S2. Sensitivity analysis of Meta. (DOCX 15 kb) [file 12955_2018_1031_MOESM2_ESM.docx]

Table S2 Sensitivity analysis of Meta

| Omitting study | COR | 95% CI | P | $I^{2}$ |
| --- | --- | --- | --- | --- |
| Chunhua, L 2013 | 0.334 | [0.316, 0.351] | <0.0001 | 97.1% |
| Nan, W 2012 | 0.299 | [0.282, 0.315] | <0.0001 | 97.2% |
| Liu, L 2016 | 0.287 | [0.270, 0.303] | <0.0001 | 96.6% |
| Qiyuan, L 2011 | 0.307 | [0.291, 0.324] | <0.0001 | 97.3% |
| Macabasco, A 2011 | 0.314 | [0.297, 0.330] | <0.0001 | 97.2% |
| Naimi, A.J 2017 | 0.308 | [0.291, 0.324] | <0.0001 | 97.3% |
| Ownby, R.L 2014 | 0.309 | [0.293, 0.326] | <0.0001 | 97.3% |
| Son, Y.J 2016 | 0.308 | [0.292, 0.324] | <0.0001 | 97.3% |
| Song, S 2017 | 0.313 | [0.297, 0.329] | <0.0001 | 97.2% |
| Wang, C 2017 | 0.280 | [0.263, 0.297] | <0.0001 | 96.5% |
| Zhang, Xuhao 2009 | 0.309 | [0.293, 0.325] | <0.0001 | 97.3% |
| Wallace, L.S 2008 | 0.309 | [0.293, 0.325] | <0.0001 | 97.3% |
| Mancuso CA 2006 | 0.301 | [0.285, 0.317] | <0.0001 | 97.1% |
| Wenning, D 2015 | 0.317 | [0.301, 0.333] | <0.0001 | 97.1% |
| Johnston, M.V 2005 | 0.308 | [0.292, 0.324] | <0.0001 | 97.3% |
| Wang, C 2015 | 0.314 | [0.297, 0.330] | <0.0001 | 97.3% |
| Halverson, J.L 2015 | 0.335 | [0.318, 0.352] | <0.0001 | 97.0% |
| Yan, Z 2012 | 0.296 | [0.279, 0.312] | <0.0001 | 96.6% |
| Husson, O 2015 | 0.297 | [0.280, 0.314] | <0.0001 | 97.3% |
